# Supplementary material for: Understanding the current and future usage of donor human milk in hospitals: An online survey of UK neonatal units
Source: Matern Child Nutr. 2023 Jul 3;19(4):e13526. doi: 10.1111/mcn.13526 (PMC10483937; doi:10.1111/mcn.13526)
Supplement: Supplementary file 1 — Supporting information. [file MCN-19-e13526-s001.docx]

**Supplemental Information**

**Supplemental Table 1:** Survey response rates from across each of the Operational Delivery Networks in the UK.

| **ODN** | **Total responses** | ***Level 1*** | ***Level 2*** | ***Level 3*** | **No. units in ODN** | **Response (%)** |
| --- | --- | --- | --- | --- | --- | --- |
| East Midlands | 4 | *0* | *2* | *2* | 11 | 36.4% |
| East of England | 14 | *3* | *6* | *5* | 17 | 82.4% |
| KSS | 7 | *3* | *2* | *2* | 16 | 43.8% |
| London | 16 | *1* | *11* | *4* | 27 | 59.3% |
| North West | 7 | *2* | *1* | *4* | 22 | 31.8% |
| Northern | 5 | *1* | *0* | *4* | 10 | 50.0% |
| Northern Ireland | 3 | *2* | *0* | *1* | 7 | 42.9% |
| Scotland | 5 | *0* | *2* | *3* | 15 | 33.3% |
| South West | 12 | *2* | *6* | *4* | 12 | 100.0% |
| Thames Valley and Wessex | 10 | *2* | *6* | *2* | 14 | 71.4% |
| Wales | 7 | *0* | *3* | *3* | 12 | 58.3% |
| West Midlands | 13 | *2* | *6* | *4* | 14 | 92.9% |
| Yorkshire and Humber | 5 | *0* | *2* | *3* | 18 | 27.8% |
| **Total** | **108** | ***18*** | ***47*** | ***41*** | **195** | **55.4%** |

**Supplemental Table 2:** Indications for the use of DHM included in enteral infant feeding protocol.

| **Indications** |  |  |  | **Yes** | **No** |
| --- | --- | --- | --- | --- | --- |
| Under a specified gestational age (weeks) | |  |  | 87 | 15 |
| Under a specific birth weight (g) |  |  |  | 77 | 25 |
| Reversed or persistently absent end diastolic flow | | |  | 56 | 46 |
| Post-medical NEC |  |  |  | 53 | 49 |
| Post-surgical NEC |  |  |  | 52 | 50 |
| Parental preference |  |  |  | 23 | 79 |
| Congenital bowel anomaly e.g., gastroschisis | | |  | 23 | 79 |
| Cardiac anomaly |  |  |  | 23 | 79 |
| Top-up (bridging) feeds |  |  |  | 22 | 80 |
| Haemodynamically unstable / inotropic support | | |  | 19 | 83 |
| HIE |  |  |  | 17 | 85 |
| PDA |  |  |  | 12 | 90 |
| Parental allergies |  |  |  | 2 | 100 |
| **Other** |  |  |  | **40** | **62** |

* Additional indications included supporting maternal efforts to establish breastfeeding, parental preference, maternal HIV, bilateral mastectomy, if a twin / triplet / quad was receiving DHM for another indication, maternal medication contraindicated to breastfeeding and consultant discretion. Several reported that parental preference was not included in the current guidance but would be included in the next.

**Supplemental Table 3:** Reported hospital usage of UK milk banks from the survey responses of hospitals that do not have a milk bank in their own hospital.

| **Milk bank** | **Number of units hospitals supplied by external milk banks^*^** |
| --- | --- |
| Hearts | 27 |
| Birmingham | 12 |
| Chester | 11 |
| Southampton | 7 |
| Oxford | 6 |
| Cambridge | 5 |
| Calderdale | 2 |
| STTH | 1 |
| St Georges | 1 |
| QCCH | 1 |
|  |  |
| **Total** | **86** |

*In addition to individual Trust milk banks which may supply hospitals within their ODN and beyond, the Scottish National Service provides equitable access to each of the 15 NNUs in all Scottish Health Boards, the South West Acute Hospital Human Milk Bank in Northern Ireland is the first line milk bank for all NNUs in Northern Ireland (n=7) and the Republic of Ireland (n=19), and the Southwest Human Milk Bank in Bristol is funded to supply all units in the southwest ODN (n = 12).
